# New Myositis Classification Criteria—What We Have Learned Since Bohan and Peter

**DOI:** 10.1007/s11926-018-0726-4

**Published:** 2018-03-17

**Authors:** Valérie Leclair, Ingrid E. Lundberg

**Affiliations:** 1 Rheumatology Unit, Department of Medicine, Solna, Karolinska Institutet, Rheumatology Unit, Karolinska University Hospital, SE-171 76 Stockholm, Sweden; 2 Rheumatology Unit, Department of Medicine, Solna, Karolinska Institutet and Karolinska University Hospital, Rheumatology Unit, Karolinska University Hospital, SE-171 76 Stockholm, Sweden

**Keywords:** Classification criteria, Inflammatory myopathy, Dermatomyositis, Polymyositis, Inclusion body myositis

## Abstract

**Purpose of Review:**

Idiopathic inflammatory myopathy (IIM) classification criteria have been a subject of debate for many decades. Despite several limitations, the Bohan and Peter criteria are still widely used. The aim of this review is to discuss the evolution of IIM classification criteria.

**Recent Findings:**

New IIM classification criteria are periodically proposed. The discovery of myositis-specific and myositis-associated autoantibodies led to the development of clinico-serological criteria, while in-depth description of IIM morphological features improved histopathology-based criteria. The long-awaited European League Against Rheumatism and American College of Rheumatology (EULAR/ACR) IIM classification criteria were recently published.

**Summary:**

The Bohan and Peter criteria are outdated and validated classification criteria are necessary to improve research in IIM. The new EULAR/ACR IIM classification criteria are thus a definite improvement and an important step forward in the field.

## Introduction

Classification criteria are meant to identify uniform and comparable groups of subjects for research purposes. In an era where international collaborations are frequent and, more than ever, needed to advance research in rare autoimmune diseases, agreement on a common set of classification criteria is critical. In 1975, Dr. Anthony Bohan and Dr. James B. Peter provided a first set of classification criteria that enabled the inflammatory idiopathic myopathy (IIM) field to evolve tremendously. They divided the IIMs into five groups: primary idiopathic polymyositis (PM), primary idiopathic dermatomyositis (DM), DM/PM associated with neoplasia, childhood DM/PM associated with vasculitis, and DM/PM with associated collagen-vascular disease [1, 2]. In the following decades, the description of new clinical subsets and a deeper understanding of IIM pathophysiology forced the myositis community to rethink and modify those criteria. Classification is not just a question of semantics [3–6], as aggregation of IIM heterogeneous subsets with different underlying molecular mechanisms likely contributes to the challenge in obtaining positive outcomes in clinical trials. An adequate IIM classification would hopefully translate in improved IIM management and outcomes. In this review, we will discuss the evolution of classification criteria since the original Bohan and Peter criteria. We will also review the recently published European League Against Rheumatism (EULAR)/American College of Rheumatology (ACR) classification criteria for adult and juvenile IIM [7].

## Classification Criteria in IIM—a Brief History

### From 1975 to 2000

In 1975, the original Bohan and Peter classification proposal for both diagnosis and classification was published and remains to this day the most widely used classification criteria in the field of IIM (Table 1). For the first time, DM and PM were differentiated by the presence of classical DM skin rashes, definitions for “definite,” “probable,” and “possible” diagnosis for each subset were proposed and importantly certain exclusion criteria to eliminate IIM mimickers were provided. Despite these novelties, the Bohan and Peter criteria were criticized for including several non-specific myositis features (i.e., myopathic electromyography), vague exclusion criteria, unclear definitions of the classical DM skin rashes, and for not specifying the exact number of features necessary to fulfill certain specific criteria (i.e., muscle biopsy). In 1977, the criteria were applied to a case series of 153 IIM subjects, but the absence of a control group precluded the authors to comment on the ability of their criteria to discriminate DM/PM from mimickers such as neuromuscular conditions [8]. Subsequent studies reported that the Bohan and Peter criteria could adequately distinguish DM/PM from systemic lupus erythematosus (SLE) and systemic sclerosis (SSc) [9]. Their combined sensitivity for definite and probable DM/PM diagnosis in 885 patients in studies published from 1977 to 1993 ranged between 74 and 100% [10].

**Table 1:** The Bohan and Peter criteria for DM and PM [1, 2]

First, rule out all other forms of myopathies
1. Symmetrical weakness, usually progressive, of the limb-girdle muscles with or without dysphagia and respiratory muscle weakness
2. Muscle biopsy evidence of myositis
Necrosis of type I and type II muscle fibers; phagocytosis, degeneration, and regeneration of myofibers with variation in myofiber size; endomysial, perimysial, perivascular, or interstitial mononuclear cells.
3. Elevation of serum levels of muscle-associated enzymes (CK, LDH, transaminases, aldolase)
4. EMG triad of myopathy
a. Short, small, low-amplitude polyphasic motor unit potentials
b. Fibrillation potentials, even at rest
c. Bizarre, high-frequency repetitive discharges
5. Characteristic rashes of dermatomyositis
Definite PM: all first four elements, probable PM: 3 of first 4, possible PM: 2 of first 4. Definite DM: rash *plus* 3 others, probable DM: rash *plus* 2 others, possible DM: rash *plus* 1 other

CK, creatinine kinase; LDH, lactate dehydrogenase; EMG, electromyography; PM, polymyositis; DM, dermatomyositis

At the time the Bohan and Peter criteria were published, a new subset of myositis resistant to corticosteroid treatment with specific tubulofilament and amyloid deposition on histopathology was described and named inclusion body myositis (IBM) [11, 12]. This subset showed distinctive clinical and laboratory features including a slowly progressive course, a specific pattern of muscle involvement, and the aforementioned typical histologic findings. This new entity was incorporated in the IIM diagnostic criteria formulated by Dalakas in 1991 [13]. These criteria, based on expert opinion, provided more detailed descriptions of each criterion of the Bohan and Peter criteria, including the histopathologic features expected to be found in DM, PM, or sporadic IBM (sIBM). Accordingly, perifascicular atrophy was considered diagnostic of DM especially if coupled with perivascular inflammatory infiltrates. Endomysial inflammation surrounding or invading non-necrotic muscle fibers without rimmed vacuoles and eosinophilic cytoplasmic inclusions was characteristic of PM.

While some investigators focused their efforts on linking histopathologic changes associated with each IIM subset to underlying pathologic mechanisms, others were discovering and describing myositis-specific autoantibodies (MSA). By 1990, seven MSA targeting different cytoplasmic ribonucleoproteins including helicase protein (Mi2), signal recognition particle (SRP), and five anti-aminoacyl-tRNA synthetases (histidyl (Jo1), threonyl (PL-7), alanyl (PL-12), glycyl (EJ), and isoleucyl (OJ)) were discovered and each one was found to be associated with unique clinical features [14, 15••]. In 1991, Love et al. proposed a novel classification of IIM based on these MSA, subsequent to a cross-sectional analysis of 212 IIM patients [16]. For each MSA subgroup, they detailed different disease profiles in terms of clinical features, human leucocyte antigen (HLA) associations, and prognosis. The “anti-synthetase syndrome” which refers to the association of interstitial lung disease (ILD), fever, arthritis, mechanic’s hand, myositis, and anti-synthetase autoantibody positivity was well-described, along with the anti-Mi2 phenotype of DM with classical rashes and good response to steroids.

In 1995, Tanimoto et al. published a new set of nine criteria to classify patients as either DM or PM based on a multicenter retrospective study with questionnaires and chart review [17]. Four of these criteria were not included in the original Bohan and Peter criteria: (1) muscle pain on grasping or spontaneous muscle pain; (2) non-destructive arthritis or arthralgias; (3) signs of systemic inflammation—fever, elevated C-reactive protein, or erythocyte sedimentation rate; and (4) the presence of anti-Jo1 antibody. Except for the latter, these new criteria were non-specific for IIM and several subsets such as juvenile IIM, cancer-associated myositis, and sIBM were not included in the analysis. A few years later, Targoff et al. proposed new modifications to the Bohan and Peter criteria with the goal of increasing the specificity of the original criteria which incorporated all the known MSAs at the time and muscle magnetic resonance imaging (MRI) [10]. sIBM was included in their IIM subsets as defined by the diagnostic criteria published by Griggs et al. that require specific clinical features and the presence on muscle biopsy of (1) inflammation, (2) vacuoles, and (3) amyloid deposits or tubulofilament by electron microscopy [18].

### From 2000 up to Now

For decades, clinicians, especially dermatologists, recognized an entity called DM *sine myositis* or amyopathic DM [19] that refers to subjects with DM rashes without evidence of muscle involvement. In 2002, Sontheimer wrote an editorial proposing the expansion of the DM spectrum to include hypomyopathic and amyopathic dermatomyositis (ADM) [20]. ADM was integrated in the 2003 revised Dalakas diagnostic criteria but since it allowed “subclinical myopathy” on investigations, ADM more closely met the definition of hypomyopathic DM introduced by Sontheimer [21]. Van der Meulen et al. emphasized the importance of histopathologic assessment in identifying IIM subsets and raised the risk of misclassification of sIBM cases as PM by previous classification criteria [22]. Their paper fueled discussions among IIM experts who questioned the currently accepted definition of PM [22–26].

The European Neuromuscular Center (ENMC) criteria designed on behalf of the Muscle Study Group and published in 2004 also required histopathologic confirmation in conjunction with clinical and laboratory criteria for IIM classification. These detailed criteria include MRI findings, some MSA, and specific immunohistochemical stainings that help differentiate DM and PM such as major histocompatibility complex class 1 (MHC-1) and membrane attack complex (MAC) [27–29]. They delineate seven IIM subsets: IBM, PM, DM, ADM, possible DM sine dermatitis, non-specific myositis, and immune-mediated necrotizing myopathy (IMNM). The IMNM subset is differentiated by histopathologic features where muscle necrosis predominates with only sparse or mild perivascular inflammatory cells and possible vascular MAC deposition. The “non-specific myositis” refers to a subset of IIM that lacks the characteristic histopathologic changes of DM, PM, or IMNM, but still presents with perivascular or perimysial inflammatory cell infiltrates or scattered endomysial CD8+ T cells not clearly surrounding or invading muscle fibers. The authors believe that these different histopathology-oriented approaches to classification better reflect the underlying pathogenic insults, suggesting DM to be a B cell-mediated microangiopathy and PM a result of a MHC-1 restricted cytotoxic T cell response. In 2011, Pestronk challenged this hypothesis and introduced a very detailed classification of myopathology for IIM based on expert opinion, setting aside the previous DM, PM, and sIBM nomenclature [30]. The six patterns constituting Pestronk’s classification are immune myopathy with perimysial pathology (IMPP), myovasculopathy, immune polymyopathy, IIM with endomysial pathology (IIM-EP), histiocytic inflammatory myopathy, and inflammatory myopathy with vacuoles, aggregates, and mitochondrial pathology (IM-VAMP). Under that classification model, a DM patient could be classified either in the IMPP or myovasculopathy group, depending on the morphological features on muscle biopsy. This proposed classification was not validated and, although histopathological features are specific for different subtypes of IIM, some IIM patients will not be classified as such because they present with limited, unspecific, or overlapping histopathological features, giving histopathology-based criteria a low sensitivity. Furthermore, histopathological features may change over time and could reflect disease duration more than clinical phenotypes in some cases.

In the first decade of the twenty-first century, several new MSAs were described including three new anti-aminoacyl-tRNA synthetases (asparaginyl (KS), tyrosyl (HA), phenylalanyl (Zo)), the anti-melanoma differentiation antigen 5 (anti-MDA5), the anti-small ubiquitin-like modifier activating enzyme (anti-SAE), the anti-transcriptional intermediary factor 1 (anti-TIF1), the anti-antinuclear matrix protein (anti-NXP-2), and the anti-3-hydroxy-3-methylglutaryl coenzyme A reductase (anti-HMGCR). Some of those autoantibodies have been linked to specific phenotypes and clinical courses such as the anti-TIF1γ, strongly associated with cancer-associated DM, or anti-HMGCR, a marker of IMNM associated with statin use [31–33]. MSAs and their role in IIM are ever-evolving topic and have been previously reviewed extensively elsewhere [14, 34–36].

In 2005, Troyanov et al. approached IIM classification from a different perspective, merging IIM clinical features with MSAs and myositis-associated autoantibodies profiles [37]. They considered overlap features, such as arthritis, ILD, or Raynaud phenomenon, as central components of their classification scheme. Their initial clinico-serological classification included four subsets: overlap myositis (OM), pure DM, pure PM, and cancer-associated myositis. The OM subset is characterized by the presence of autoantibodies such as anti-synthetases, SSc-associated autoantibodies, anti-SRP, and/or overlap features. Pure DM refers to the presence of a DM rash and/or positivity for anti-Mi2, and pure PM includes patients without overlap features or autoantibodies. Using this purely clinical approach, OM was the most frequent subset in the 100 IIM subjects included in their study. This subgroup also showed good response to corticosteroids. This clinico-serological classification was correlated with the muscle biopsies of 178 IIM patients based on the ENMC histopathologic criteria, and both approaches were found to be complementary [38••]. This classification was later updated by the authors to integrate newly described MSAs such as anti-MDA5, and more subsets namely overlap myositis with DM features (OMDM), necrotizing autoimmune myositis (NAM), and sIBM. Nailfold capillaroscopy abnormalities were also introduced as a characteristic feature of DM and OM [39••, 40]. In 2016, Benveniste et al. outlined a classification scheme integrating clinical and pathological features with four IIM subsets (IBM, OM, IMNM, and DM) and subdivision by MSA status [15••]. This interesting classification that bears similarities with the clinico-serological approach proposed by Troyanov et al. is however based on expert opinions and requires further validation.

### The EULAR/ACR Classification Criteria

**Fig. 1:**
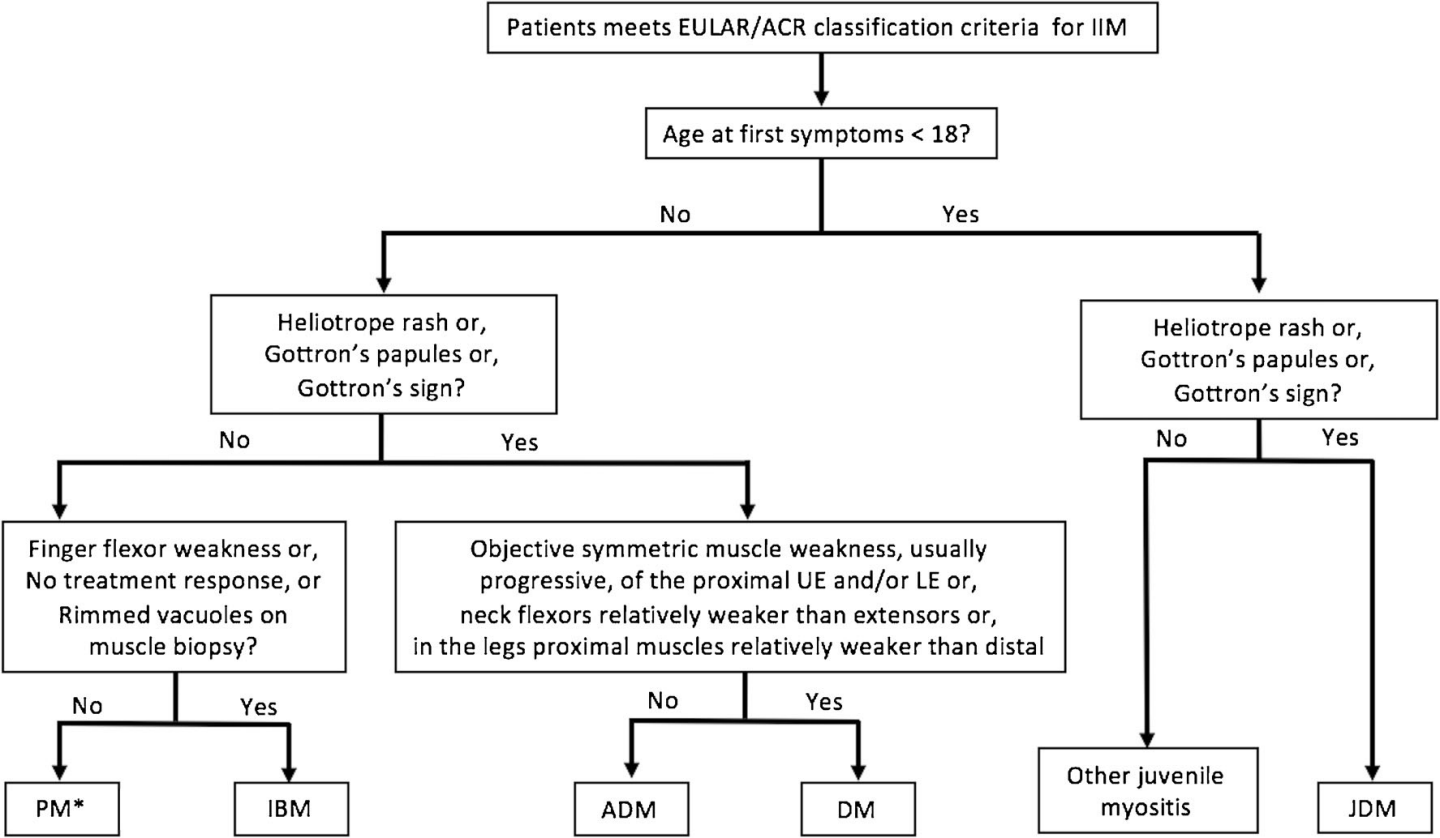
Subgroups of IIM according to the 2017 EULAR/ACR classification criteria [7]. *The PM subset includes immune-mediated necrotizing myopathies (IMNM). PM, polymyositis; IBM, inclusion body myositis; ADM, amyopathic dermatomyositis; DM, dermatomyositis; JDM, juvenile dermatomyositis

IIM classification has been a matter of debate for decades, and myositis experts agree on the importance of reaching a consensus to improve future clinical trials. The previous classification criteria were mainly empirically derived and not properly validated. When studies were based on patient’s data, patient cohorts were often small, single-center, and did not include controls. To palliate for this, a group of international IIM experts including adult and pediatric rheumatologists, neurologists, dermatologists, epidemiologists, and biostatisticians was assembled in 2004 to develop new IIM classification criteria following the recommendations published by the EULAR/ACR [3, 4]. The goal was the development of classification criteria that would distinguish IIMs from mimickers and categorize IIMs in major subgroups with a minimum of clinical and readily available laboratory features. A consensus method was used for study design, definition of possible criteria candidates, and selection of IIM subgroups and comparators. Ninety-three variables were identified, selected from both previous criteria and suggestions of experts, and applied to 976 IIM cases and 624 non-IIM cases from 47 centers across the world. Two models, with or without muscle biopsy results, were developed to better reflect some clinical settings such as pediatrics, where performing muscle biopsy is not standard of care. Based on a complex but robust method, 16 variables deemed to provide better discrimination for IIM cases were weighted and included in a final criteria set presented in Table 2 and Fig. 1. A web-based calculator provides a score that represents the probability of a particular individual to have an IIM. Thus, researchers using this classification retain the capability to tailor the inclusion criteria to a certain level of sensitivity or specificity depending on the type of study considered. It provides detailed definition for each criteria and showed a sensitivity and specificity for a probable IIM diagnosis of 93 and 88%, respectively, when biopsy results are included (Table 3) [7, 41]. As with any classification schema, the EULAR/ACR model presents limitations. IIM being a heterogeneous group of diseases, not enough patients from rare subgroups (e.g., IMNM, hypomyopathic DM, and juvenile PM) could be recruited and only one MSA, anti-Jo1, was documented in enough subjects to be included in the final variables. It is plausible that with wider use of myositis antibodies, anti-Jo1 could be replaced by any MSA. Muscle MRI, only available in 38% of cases, was also excluded from the analysis. Thus, these criteria will soon require revision using a cohort with further data on MSAs and MRI, as well as validation in an external cohort with IIM cases and comparators.

**Table 2:** Components of the 2017 EULAR/ACR classification criteria for adult and juvenile IIM

When no better explanation for the symptoms and signs exists these classification criteria can be used		
Variable	Score	
	No muscle biopsy	With muscle biopsy
Age of onset of first symptom assumed to be related to the disease ≥ 18 and < 40 years	1.3	1.5
Age of onset of first symptom assumed to be related to the disease ≥ 40 years	2.1	2.2
Muscle weakness		
Objective symmetric weakness, usually progressive, of the proximal upper extremities	0.7	0.7
Objective symmetric weakness, usually progressive, of the proximal lower extremities	0.8	0.5
Neck flexors are relatively weaker than neck extensors	1.9	1.6
In the legs, proximal muscles are relatively weaker than distal muscles	0.9	1.2
Skin manifestations		
Heliotrope rash	3.1	3.2
Gottron’s papules	2.1	2.7
Gottron’s sign	3.3	3.7
Other clinical manifestations		
Dysphagia or esophageal dysmotility	0.7	0.6
Laboratory measurements		
Anti-Jo1 autoantibody present	3.9	3.8
Elevated serum levels of CK or LDH^*^ or ASAT/AST/SGOT^*^ or ALAT/ALT/SGPT^*^	1.3	1.4
Muscle biopsy features—presence of:		
Endomysial infiltration of mononuclear cells surrounding, but not invading, myofibres		1.7
Perimysial and/or perivascular infiltration of mononuclear cells		1.2
Perifascicular atrophy		1.9
Rimmed vacuoles		3.1

Modified from [7]

Anti-Jo1, anti-histidyl-tRNA synthetase; CK, creatine kinase; LDH, lactate dehydrogenase; ASAT/AST/SGOT, aspartate aminotransferase; ALAT/ALT/SGPT, alanine aminotransferase

**Table 3:** Comparison of various classification and diagnostic criteria sets

	Type of criteria	SN^a^	SP^a^	Type of classification				IIM subtypes proposed
				EMG	BX	MSA	MRI	
Bohan and Peter [1, 2]	Diagnostic/classification	94–98%	29–55%	X	X			DM, PM Childhood DM/PM with vasculitis DM/PM with neoplasia or CTD.
Tanimoto [17]	Classification	89–96%	29–31%	X	X	X		DM, PM
Targoff [10]	Diagnostic/classification	97–93%	29–89%	X	X	X	X	DM, PM Childhood DM/PM with vasculitis DM/PM with neoplasia or CTD IBM
Dalakas [21]	Diagnostic	6–77%	99%	X	X			DM, PM, ADM
ENMC [27]	Classification	52–71%	82–97%	X	X	X	X	DM, PM, ADM DM sine dermatitis Non-specific myositis IMNM IBM
EULAR/ACR [7]	Classification	No biopsy			X^b^	X		DM, PM, ADM JDM IBM
		87%	82%					
		Biopsy						
		93%	88%					

SN, sensitivity; SP, specificity; EMG, electromyography; BX, muscle biopsy; MSA, myositis-specific autoantibodies; MRI, magnetic resonance imaging; DM, dermatomyositis; PM, polymyositis; ADM, amyopathic dermatomyositis; CTD, connective tissue disease; IBM, sporadic inclusion body myositis; IMNM, immune-mediated necrotizing myopathy; JDM, juvenile dermatomyositis

^a^“Definite” and “probable” diagnoses were considered positive cases, and “possible” diagnoses, negative cases. Specialist diagnosis represented the gold standard [7, 41]

^b^The EULAR/ACR classification can be used with or without muscle biopsy results

## Discussion

Classification of IIM has been and will likely remain the topic of much discussion and debate. Attempts at categorizing a heterogeneous group of diseases with multiple possible organ involvement, considerable overlapping features, and different underlying pathologic molecular mechanisms that are yet to be fully deciphered are a daunting task. In addition, some IIM subsets such as sIBM are characterized by an insidious onset with characteristic histologic findings appearing late in the disease course, which may lead to misclassification of affected patients as PM in the early phase of their disease [42•]. More clinical sIBM diagnostic criteria have been recently proposed to address this issue [43, 44]. Certain subsets of the IIM disease spectrum can be dominated by extra-muscular features with no or mild muscle weakness which presents yet another challenge for IIM classification. The anti-synthetase syndrome which encompasses a large spectrum of clinical presentations influenced, at least in part, by the detected anti-synthetase autoantibody is a good example of this [45–47]. According to the new EULAR/ACR criteria, this subset still falls in the PM category, which may seem outdated given the subset’s known distinct phenotype and clinical course [48, 49]. In that regard, a clinico-serological approach seems better suited.

A careful evaluation of the clinical features of IIM including muscle weakness pattern is also central to classification in a research setting. When assessing muscle weakness, clinicians should be aware of the caveats of frequently used tools, such as the ceiling effect of the MMT8 [50•]. As diagnostic methods are constantly evolving, classification criteria need to be periodically reviewed to leverage new diagnostic capabilities. Muscle MRI has been incorporated in some of the recent criteria set. This technique allows for assessment of muscular inflammation in terms of extent and localization [51•, 52•, 53]. Atrophy and fatty replacement of muscle tissue, a reflection of disease damage, can also be assessed. However, muscle MRI findings are not specific for IIM and intramuscular edema can be found in several other disorders including trauma, myonecrosis, infection, rhabdomyolysis, and non-inflammatory myopathies [53]. Accessibility might be an issue with this imaging technique, which can also be the case of MSA detection, even in this era of commercial MSA assay availability. One also needs to keep in mind that individual MSA have a low sensitivity for IIM, although collectively their sensitivity may reach 60–70%, and therefore their absence do not exclude an IIM diagnosis [54]. On the other hand, the presence of MSA provides precious insights to clinicians as previously stated, and future large-scale epidemiologic studies will certainly help integrate MSA in a valid classification scheme. Of note, even if EMG results were excluded from new EULAR/ACR criteria, this technique remains an important adjunct to IIM diagnosis and should be ordered especially in cases where an alternate neuromuscular diagnosis is suspected. As suggested by Troyanov et al., nailfold capillaroscopy is also a safe, non-invasive procedure that provides valuable information to the clinician. However, the ability of capillaroscopy to discriminate DM and PM cases, which are frequently merged together in studies, has yet to be established, and its’ evaluation and interpretation are not standardized [55]. Whether the new 2017 EULAR/ACR classification criteria for myositis will be useful to identify patients with shared molecular pathophysiology has still to be tested.

## Conclusions

Over the years, many IIM experts have offered invaluable contributions to our knowledge of the IIM disease spectrum and have proposed various approaches to its classification. Bohan and Peter criteria continue to be used widely in medical schools and residency programs and still provide deep understanding of IIM to the trainees. In the hope of transitioning to a more contemporary classification, the EULAR/ACR criteria have been developed and validated with a robust methodology, an improvement over previous criteria sets. The new IIM classification criteria are a valuable addition to the myositis disease activity core measures endorsed by IMACS and the recently published ACR/EULAR adult PM/DM and juvenile DM response criteria [50•, 56, 57••, 58••]. In the future, these new classification and response criteria should continue to evolve and reflect epidemiologic and molecular knowledge advances on IIM. Future clinical trials are expected to use these new criteria hopefully leading to novel therapeutics in myositis.
